# Development and Biological Evaluation of *Aronia melanocarpa* Extract-Loaded Gelatin–Collagen Nanofibers on B16F10 Cell

**DOI:** 10.3390/polym18151911

**Published:** 2026-08-04

**Authors:** İrem Gün, Rukiye Yiğit, Filiz Altay, Büşra Yusufoğlu

**Affiliations:** 1 Department of Chemistry, Faculty of Science and Letters, Istanbul Technical University, Ayazağa Campus, 34469 Istanbul, Türkiye; gun24@itu.edu.tr; 2 Department of Food Engineering, Faculty of Chemical and Metallurgical Engineering, Istanbul Technical University, Maslak, 34469 Istanbul, Türkiye; yigitru@itu.edu.tr (R.Y.); lokumcu@itu.edu.tr (F.A.)

**Keywords:** *Aronia melanocarpa*, nanofiber encapsulation

## Abstract

Background: *Aronia melanocarpa* is a sustainable source of phenolic compounds and anthocyanins with antioxidant and biological activities; however, the instability of these compounds limits their application. This study aimed to develop gelatin–collagen electrospun nanofibers loaded with *Aronia melanocarpa* extract (AME) to improve phytochemical stability. Methods: AME-loaded nanofibers were produced by electrospinning and evaluated for their physicochemical properties, antioxidant activity, antimicrobial effects, antiproliferative potential, and molecular interactions with epidermal growth factor receptor (EGFR). Results: AME incorporation increased the viscosity from 0.66 to 1.54 Pa·s and the fiber diameter from 0.39 to 0.84 μm while reducing the contact angle from 39.71° to 28.83°. The total phenolic content increased from 19.365 to 48.775 mg GAE/g, and the antioxidant capacity increased from 2.852 to 7.880 mg TE/g. At 500 μg/mL, AME reduced the maximum growth rate of *S. aureus* from 0.227 to 0.121 OD600/h and prolonged the lag phase, indicating a 53% bacteriostatic effect. While Cyanidin-3-O-glucoside (Cy3G) was the dominant anthocyanin, cyanidin showed the highest retention rate after electrospinning, and cyanidin-3-rutinoside demonstrated strong interaction with EGFR (−9.0 kcal/mol). Conclusions: AME-loaded nanofibers are potential sustainable products for health applications.

## 1. Introduction

Melanoma is considered one of the most aggressive forms of cancer and poses a significant clinical problem because of its high metastatic potential and the development of resistance to conventional chemotherapeutic agents [1]. Therefore, in recent years, the anticancer and antiproliferative potential of plant-derived bioactive compounds has been intensively investigated. The use of natural compounds is considered an important alternative approach, particularly for reducing the toxic effects that synthetic drugs may cause [2]. In this context, *Aronia melanocarpa* L. (black chokeberry) stands out as an important functional food source for health because it is rich in phenolic compounds. This fruit, belonging to the Rosaceae family, contains numerous phenolic compounds, such as proanthocyanidins, anthocyanins, hydroxycinnamic acids, flavonol glycosides, cyanidin glycosides, and quercetin glycosides, and the composition of these compounds can vary depending on the extraction method and solvents used. Aronia berries, particularly those known for their high polyphenol, flavonoid, and anthocyanin contents, are considered one of the richest natural sources of this type of nutrient [3,4]. The rich phytochemical profile of aronia berries protects cells against oxidative damage by scavenging free radicals, and these berries exhibit powerful biological effects capable of inhibiting cancer cell proliferation [5]. Furthermore, these compounds are reported to have therapeutic effects on skin disorders, inflammation, and various chronic diseases. These biological effects occur through mechanisms such as scavenging free radicals, inhibiting reactive oxygen species (ROS) and reactive nitrogen species (RNS), reregulating antioxidant enzyme activity, suppressing pro-oxidants, and modulating cellular signaling pathways [6].

Aronia, a byproduct of fruit, contains a significant portion of the total phenolic compounds and dietary fiber [7]. Aronia fiber, obtained from this pulp, is considered a strategic resource for sustainable waste management and the conversion of agricultural byproducts into value-added products [8]. Furthermore, fermentation or purification of aronia can further enhance the functional properties and biological activities of the resulting products [9]. However, the chemical structure of anthocyanins and other phenolic compounds found in aronia is highly sensitive to environmental factors such as light, temperature, pH changes, and oxidation [10]. This limits the stability and bioavailability of these bioactive compounds, making their direct therapeutic use difficult. Therefore, increasing the stability of bioactive components is crucial. The encapsulation technologies developed for this purpose stand out as important solutions. Encapsulation systems enable the effective transport of bioactive substances to target tissues by protecting them from external environmental influences [11,12].

At this point, nanofiber technologies offer a promising approach to increase the stability of bioactive components. Nanofibers are nanostructures that provide significant advantages in encapsulation applications because of their nanometer-scale diameters, high surface area/volume ratios, and tunable porous structures [13]. Electrospinning, one of the most commonly used methods in nanofiber production, enables the development of stable and biocompatible carrier systems by encapsulating active components within a polymeric matrix [14]. Currently, electrospinning technology is widely used in many biomedical applications, such as controlled drug release systems, tissue engineering scaffolds, and the development of wound healing biological dressings [6]. Nanofibers produced via this method can increase therapeutic efficacy by providing controlled release of bioactive components while minimizing systemic toxicity [15]. Furthermore, the fiber diameter and morphology can be precisely controlled by optimizing parameters such as the voltage, flow rate, and polymer concentration during the electrospinning process [16]. However, in the literature, aronia has been studied for its high anthocyanin content and bioactive effects, particularly in the production of various films, including pH-sensitive smart packaging [17,18,19,20,21]. However, research has also been conducted on its use in various other fields, such as bioelastomers in food packaging [22], carbon nanosheets in drug delivery [23] and the production of polyurethane nanofibers [24]. Although numerous studies exist in the literature on the antioxidant and biological activities of aronia berries, studies on the utilization of fibers obtained from aronia in nanofiber structures via the electrospinning method are quite limited. Therefore, this study aims to use aronia fibers as both a structural support and a bioactive source in nanofiber systems. In this context, the encapsulation of aronia into gelatin and collagen polymers is an innovative approach for this purpose. The hydrophilic biopolymer gelatin is a form of protein that is formed by the hydrolysis of collagen [25]. Gelatin and collagen are highly functional natural polymers due to their nontoxicity, cost-effectiveness, biocompatibility, and biodegradability [26] and are widely used in clinical applications—particularly in cosmetics and food—as pharmaceutical products, binders, and wound dressings [25]. These developed bionanocomposite structures aim to combine the strong antioxidant potential of aronia berries and the stability advantages offered by nanofiber technology, thereby presenting a natural and nanotechnology-based approach for the treatment of skin cancer (melanoma). This approach is expected not only to increase the biological activity of cancer cells but also to contribute to the development of more stable and long-lasting biomaterials by protecting sensitive bioactive components. Furthermore, the findings provide an important scientific basis for the future development of wound dressings and targeted drug delivery systems. Electrospun nanofibers are also considered promising biomaterials for wound dressing and skin tissue engineering, and evaluating their antibacterial activity against wound-associated pathogens is important in addition to assessing their antiproliferative potential. Therefore, antibacterial activity against the methicillin-resistant *Staphylococcus aureus* (MRSA) substrain COL was investigated to assess the multifunctional properties of the developed AME-loaded nanofibers.

To date, no studies have directly reported electrospun fibers loaded with *Aronia melanocarpa* extract (AME) tested on B16F10 melanoma cells. However, Aronia is a potent, formulatable polyphenol source, electrospun nanofibers are proven carriers for anticancer agents, and B16F10 is a standard melanoma model. Combining these three elements appears to be an open and plausible research opportunity.

## 2. Materials and Methods

### 2.1. Materials

AME was purchased from Dr. Aronia from Istanbul, Turkey. Commercially available AME contains a high amount of dietary fiber (56.2 g/kg) according to the nutritional values stated on the package and is rich in antioxidant components such as β-carotene (7706 mg/kg), β-cryptoxanthin (4.63 mg/kg), vitamin C (137 mg/kg), and tocopherols (17.1 mg/kg). The extract also contains various B vitamins and minerals, such as potassium (2180 mg/kg), calcium (322 mg/kg), magnesium (162 mg/kg), iron (9.3 mg/kg), and zinc (1.47 mg/kg). In this work, gelatin and collagen were used as the main polymer matrix. Fifty percent acetic acid was used as the solvent. Aronia berry powder was added as a functional component. A brief summary is given in the graphical abstract.

#### 2.1.1. Chemicals

Beef gelatin (220 bloom) and collagen were obtained from Alfasol and Rotel (İstanbul, Türkiye), respectively. Folin–Ciocalteu reagent, standards of gallic acid and Trolox, and sodium carbonate were obtained from Sigma–Aldrich (Steinheim, Germany). DPPH (2,2-difenil-1-pikrilhidrazil) was supplied by TCI (Tokyo, Japan). Acetic acid and methanol were obtained from Merck and Chemsolute (Th. Geyer, Renningen, Germany), respectively.

#### 2.1.2. Cell Culture

The B16F10 (cutaneous melanoma) cell line was plated in 96-well plates at a density of 2 × 10^5^ cells per well in DMEM (Dulbecco’s modified Eagle’s medium) supplemented with glucose, 10% FBS (Ebsdorfergrund, Germany) and 1% penicillin–streptomycin (Gibco Waltham, MA, USA). When the cells reached a density of 70%, the aronia extract was diluted at ratios of 1/2, 1/10, 1/20, 1/50 and 1/100, fibers and aronia-containing fibers were added to the cells, and the cells were incubated in medium containing 5% CO_2_ at 37 °C for 24–48 h. After 24 and 48 h, the cells were subjected to the MTT (0.35 mg/mL) test. After 3 h of incubation, the medium was removed, and 100 µL of DMSO was added. Cell viability was measured at 570 nm after 30 min of incubation, and the IC50 value was calculated.

(1) $$Formula=Test-\frac{negative\;control}{positive\;control}\times 100$$

### 2.2. Methods

#### 2.2.1. Preparation and Characterization of Feed Solution

The feed solution for the electrospinning process, containing 20% gelatin (*w*/*v*) and 20% collagen (*w*/*v*), was prepared in 50% (*v*/*v*) acetic acid. Aronia powder was then added to the solution in an amount corresponding to 10% of the total polymer weight. The solutions were homogenized with a magnetic stirrer at 60 °C at 700 rpm for 30 min (Table 1).

The electrical conductivity of the feed solutions was measured by a conductometer (WTW LF95, Weilheim, Germany).

A rheometer (Haake Rheostress 1, Thermo Fisher Scientific, Karlsruhe, Germany) with a plate–plate sensor (dia = 35 mm, gap = 1 mm) was used to measure the viscosity of the feed solutions at 23 °C. The shear rate was increased from 0.001 to 200 s^−1^ over 120 s. The data were modeled with the power-law equation by using the software of the rheometer (Rheowin, Germany) as follows:

(2) $${\tau =\kappa \cdot \dot{\gamma }}^{n}$$

where τ is the shear stress (Pa), K is the consistency index (Pa·sn), n is the flow behavior index and $\dot{\gamma }$ is the shear rate (s^−1^). The apparent viscosity (η) was calculated via the following equation:

(3) $${\eta =\kappa \cdot \dot{\gamma }}^{n-1}$$

The maximum shear rate was calculated according to Equation (4), which is suitable for materials with power-law properties in a tubular geometry:

(4) $${\dot{\gamma }}_{max}=\left(\frac{3n+1}{4n}\right)\frac{4Q}{\pi R^{3}}$$

where Q is the volumetric flow rate of the material and R is the inside radius of the tube. The radius of the needle (inner diameter of the 17-G needle is 1.067 mm) where the feed solution exits and is exposed to the electric field was 0.534 × 10^−3^ m. The flow rate of the feed solution for this study was 0.6 mL/h, which is equal to Q = 1.667 × 10^−10^ m^3^ s^−1^. The maximum shear rates were subsequently calculated according to Equation (4). The apparent viscosities were calculated from Equation (3) at the corresponding maximum shear rate (MSR) values [27].

#### 2.2.2. Electrospinnig

The electrospinning process was carried out with equipment (Inovenso NE100, Istanbul, Turkey) consisting of a syringe pump (New Era Pump Systems Inc., NE-300, New York, NY, USA), the tip of the needle where the voltage was applied, the collector plate and the high-voltage power supply (Nanofen, Ankara, Turkey). The electrospinning parameters were optimized as follows: flow rate, 0.6 mL/h; distance between the needle and collector, 15 cm; and voltage, 15 kV.

#### 2.2.3. Preparing Extracts of Nanofibers

The extraction of samples for total phenolic content, antioxidant activity, and individual phenolic compound analyses was performed according to previously described methods. Five milliliters of 75% methanol with 0.1% formic acid was added to each 1 g sample and sonicated for 15 min. The supernatant was collected after centrifugation (NuAire, Hitachi, Plymouth, MN, USA) at 2500 rpm for 10 min. Another 5 mL of 75% methanol was added to the residue, and the extraction step was repeated. Both supernatants were collected in another test tube and adjusted to a final volume of 10 mL. The resulting supernatants were stored at +4 °C for later use [28].

#### 2.2.4. Spectrophotometric Assays

The total phenolic content (TPC) of the extract was determined according to a previously described method [29]. The results are expressed as mg of gallic acid equivalents (GAE) per 100 g of dry weight. The total antioxidant capacity was determined via two different assays. Copper (II)-reducing antioxidant capacity (CUPRAC) and 2-diphenyl-1-picrylhydrazyl (DPPH) assays. The results are expressed as mg of Trolox equivalents (TE) per 100 g of dry weight [30,31]. Total anthocyanin content (TAC) characterization and measurements were carried out via UV–visible spectroscopy according to the method. Buffer solutions of 0.025 M KCl at pH 1 and 0.4 M sodium acetate at pH 4.5 were prepared, and quantitative analysis was performed on the basis of the difference in absorbance [32].

Thirty milligrams of nanofibers were weighed and mixed with 1 mL of distilled water. The mixture was centrifuged at 5000 rpm for 10 min. After centrifugation, the supernatant was used for phenolic and antioxidant analysis. The total phenolic content was determined via the Folin–Ciocalteu method [29]. Gallic acid solutions in the range of 5–500 ppm were prepared as standards, and the calibration curve equation was y = 0.2761 + 0.0074x, R^2^ = 0.9445. For the analysis, 250 µL of the sample was mixed with 1250 µL of Folin–Ciocalteu reagent and 1000 µL of sodium carbonate solution. The mixture was incubated in the dark for 2 h. The spectrophotometric measurements were performed at 765 nm, and the results are presented as mg gallic acid equivalents (GAE)/g nanofiber. Antioxidant activity was evaluated via the DPPH assay [33]. Trolox solutions (1–500 ppm) were used as standards, and the corresponding calibration curve equation was y = −0.0407 + 0.0024x, R^2^= 0.9994. Fifty microliters of sample was mixed with 1950 µL of DPPH solution. The mixture was incubated in the dark for 30 min. The absorbance was measured at 515 nm, and the results are presented as mg Trolox equivalents (TE)/g nanofiber.

#### 2.2.5. Analysis of the Anthocyanin Profile via HPLC

The anthocyanin profiles of the samples were identified via an HPLC system (Shimadzu Corp., Kyoto, Japan) equipped with a pump (LC-20AD), DAD detector (SPDM20A), autosampler (SIL20A HT), column oven (CTO-10ASVP), degasser (DGU-20A5R), and communication bus module (CMB-20A) according to [34]. Solutions A (7.5% formic acid in acetonitrile) and B (7.5% formic acid in deionized water) constituted the mobile phase. The separation was carried out using an Intersil^®^ ODS column (C18, 250 mm × 4.6 mm length and 5 μm particle size; GL Sciences, Tokyo, Japan). The chromatograms were recorded at 520 nm, the flow rate was 1 mL/min, and the column oven temperature was 20 °C. After passing through a 0.20 μm filter, the samples were injected (20 µL) onto an HPLC.

Kuromanin (cyanidin-3-O-glucoside chloride), callistephin (pelargonidin-3-O-glucoside chloride), cyanin (cyanidin-3,5-di-O-glucoside), malvidin (malvidin-3-O-glucoside chloride), keracyanin (cyanidin-3-O-rutinose chloride), delphinidin (delphinidin 3-O-β-D-glucoside chloride), and peonidin (peonidin 3-O-glucoside chloride) standards were used to determine the anthocyanin profiles of the samples, and the anthocyanin concentrations are reported in mg/100 g.

#### 2.2.6. Characterization of the Electrospun Nanofibers

*Scanning electron microscopy*

Morphological analyses of the NFs were conducted by scanning electron microscopy (SEM) (Zeiss EVO LS 10, Baden-Württemberg, Germany). The average fiber diameter was calculated from 100 randomly selected fibers via ImageJ version 1.54g software (NIH, Bethesda, MD, USA).

*Contact angle measurements*

The contact angles of the membranes were measured on a KSV Attension Theta contact angle device using the scissile drop mode on dried membranes. A small droplet of distilled water was delivered onto the membrane surface, which was left for 5 s, and then the image of the droplet on the membrane surface was taken at 10 frames and 1 s intervals. To ensure the accuracy of the measurement, the analyses were performed at 4 different locations on the membranes [35].

*FTIR measurements*

FTIR measurements were performed on samples of aronia and aronia–anthocyanin interactions via an FTIR Tensor II spectrophotometer (Bruker Scientific LLC, Billerica, MA, USA) according to the methods of [36]. Measurements of the powdered samples were carried out in the frequency range of 400–4000 cm^−1^. Band components were determined on the basis of their average frequency values.

*Cavity detection guided blind docking of anthocynins and receptors in B16F10 Cells*

Molecular docking is a widely used computational approach to predict the preferred binding orientation of a ligand at a protein binding site and to evaluate the stability of the resulting protein-ligand complex. In this study, epidermal growth factor receptor (EGFR), one of the most important receptors in melanoma cancer, was selected. For molecular docking studies targeting the EGFR kinase domain, the crystal structure of EGFR (PDB ID: 2GS2) was obtained from the RCSB Protein Data Bank. As ligands, cyanidin-3-glucoside (PubChem ID: 441667), cyanidin-3-arabinoside (PubChem ID: 91810602), cyanidin-3-rutinoside (PubChem ID: 441674), and cyanidin-3-galactoside (PubChem ID: 176457), found in AME, were obtained from the PubChem database and used. Blind molecular docking was performed using the CB-Dock2 server. The loaded protein structure was automatically prepared by the server. Potential ligand-binding cavities were identified using the CurPocket algorithm. For each predicted cavity, docking positions and corresponding binding affinities (kcal/mol) were automatically generated. Docking results were evaluated based on binding affinity, cavity volume, and ligand–protein interaction patterns. The binding position with the lowest binding energy and biologically relevant interactions was selected for further analysis and visualization [37].

#### 2.2.7. MTT Cell Viability Analysis of the Nanofibers

The B16F10 (cutaneous melanoma) cell line was plated in 96-well trays containing Dulbecco’s modified Eagle’s medium (DMEM) containing concentrated glucose, 10% FBS, and 1% penicillin–streptomycin at a density of 2 × 10^4^ cells per well. When the cells reached 70% confluence, the aronia extract was diluted to 1/2, 1/10, 1/20, 1/50, and 1/100, and fibers and aronia-containing fibers were added to the cells. The cells were then incubated at 37 °C with 5% CO_2_ for 24 to 48 h. After 24 and 48 h, the cells were subjected to an MTT test (0.35 mg/mL). After 3 h of incubation, the medium was removed, and 100 mL of DMSO was added. Cell viability was assessed at 570 nm after 30 min of incubation, and the IC50 value was calculated [38]. Untreated B16F10 cells cultured in complete DMEM served as the negative control. The cells treated with 0.1% DMSO, corresponding to the highest solvent concentration used in the treatment groups, served as the vehicle control. Blank wells containing culture medium and MTT reagent without cells were included for background correction, and their absorbance values were subtracted from the corresponding experimental wells before data analysis. The positive control wells contained untreated B16F10 cells cultured in complete DMEM and presented 100% cell viability. The negative control wells contained complete culture medium without cells and were used for background correction. The cells treated with 0.1% DMSO, corresponding to the highest solvent concentration used in the treatment groups, served as the vehicle control. Background absorbance from the negative control wells was subtracted from all experimental measurements before calculating cell viability. In this study, B16F10 cells were selected because they represent a well-established and widely used in vitro model for melanoma research, particularly for the preliminary evaluation of their antiproliferative activity.

Formula = Test − Negative control/Positive control × 100(5)

#### 2.2.8. Growth Curve Assay

The antibacterial activity of AME was evaluated against the methicillin-resistant *Staphylococcus aureus* (MRSA) substrain COL by monitoring bacterial growth curves. A single colony was inoculated into tryptic soy broth (TSB) and incubated at 37 °C under aerobic shaking conditions until the exponential growth phase was reached. The bacterial suspension was then exposed to AME at final concentrations of 10, 20, 50, 100, and 500 μg/mL. Untreated bacterial cultures served as the growth control, while culture medium without bacteria was used as the blank for background correction. The optical density was measured at 600 nm (OD600) every hour for 10 h via a microplate reader. Growth curves were generated from the corrected OD600 values, and the maximum growth rate (Vmax) was calculated from the exponential phase of each growth curve. Each experiment was performed in triplicate, and the results are presented as the means ± standard deviations. Statistical analysis was performed via IBM SPSS Statistics Version 22, and differences among groups were evaluated via the independent *t* test, with *p* < 0.05 considered statistically significant [39].

#### 2.2.9. Statistical Analysis

Statistical analysis of the results was performed via IBM SPSS Statistics Version 22, and an independent *t* test was applied to compare the differences between the means of the solutions and the nanofibers themselves. In terms of the MTT results, among the 24 h groups, only NF significantly decreased cell viability in a dose-dependent manner (R^2^ = 0.90, *p* = 0.013). At 24 h, there was a borderline trend (*p* = 0.076), whereas at 24 h, AM-NF showed no significant dose–response relationship. For each anthocyanin, a one-way ANOVA was performed between the AME and AME-loaded NF groups (Python (3x) /scipy.stats.f_oneway, *n* = 3/group). Significance levels: * *p* < 0.05, ** *p* < 0.01, *** *p* < 0.001.

## 3. Results

### 3.1. Properties and Electrospinnability of the Feed Solutions

The composition, electrical conductivity and rheological properties of the feed solutions are shown in Table 1. The production of nanofibers requires a low electrical conductivity. However, solutions with zero conductivity cannot be electrospun into nanofibers. The electrical conductivities of the solutions were 2.87 ± 0.15 mS/cm and 2.69 ± 0.07 mS/cm for the neat and aronia-loaded solutions, respectively. The viscosities of the neat and aronia-loaded nanofibers were 0.66 ± 0.06 Pa·s and 1.54 ± 0.29 Pa·s, respectively (*p* < 0.05), and the viscosity of the solution and the diameter of the nanofibers increased, which was sufficient for nanofiber formation, as confirmed by SEM images. These results indicate that the samples have electrospinning capabilities. Viscosity also plays an important role in fiber characterization; specifically, bead formation is directly related to the solution concentration. As the solution concentration increases, the viscosity also increases. Since continuous fibers cannot be produced from a low-viscosity solution, at high viscosities, the electrical charges cannot provide sufficient force to form the fiber structure, and the solution viscosity is one of the key parameters for a successful electrospinning process.

### 3.2. Morphology of the Electrospun Samples

**Figure 1:**
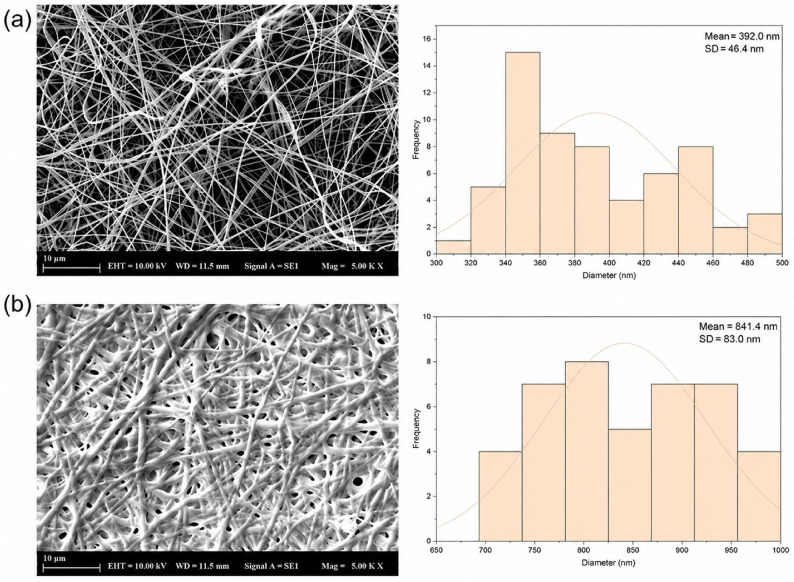
SEM images and diameter distributions of (**a**) neat and (**b**) aronia-loaded nanofibers.

SEM images of the neat and aronia-loaded nanofibers are shown in Figure 1. Bead-free and uniform nanofibers were obtained under the applied electrospinning conditions. However, the fiber diameter is directly influenced by the viscosity of the solution. The nanofiber diameter was 392.0 ± 46.4 nm for neat NF and 841.4 ± 83.0 nm for aronia-loaded nanofibers (*p* < 0.05), and the viscosity of the solution and the diameter of the nanofibers increased significantly during the aronia encapsulation process.

### 3.3. FT-IR Spectroscopy

**Figure 2:**
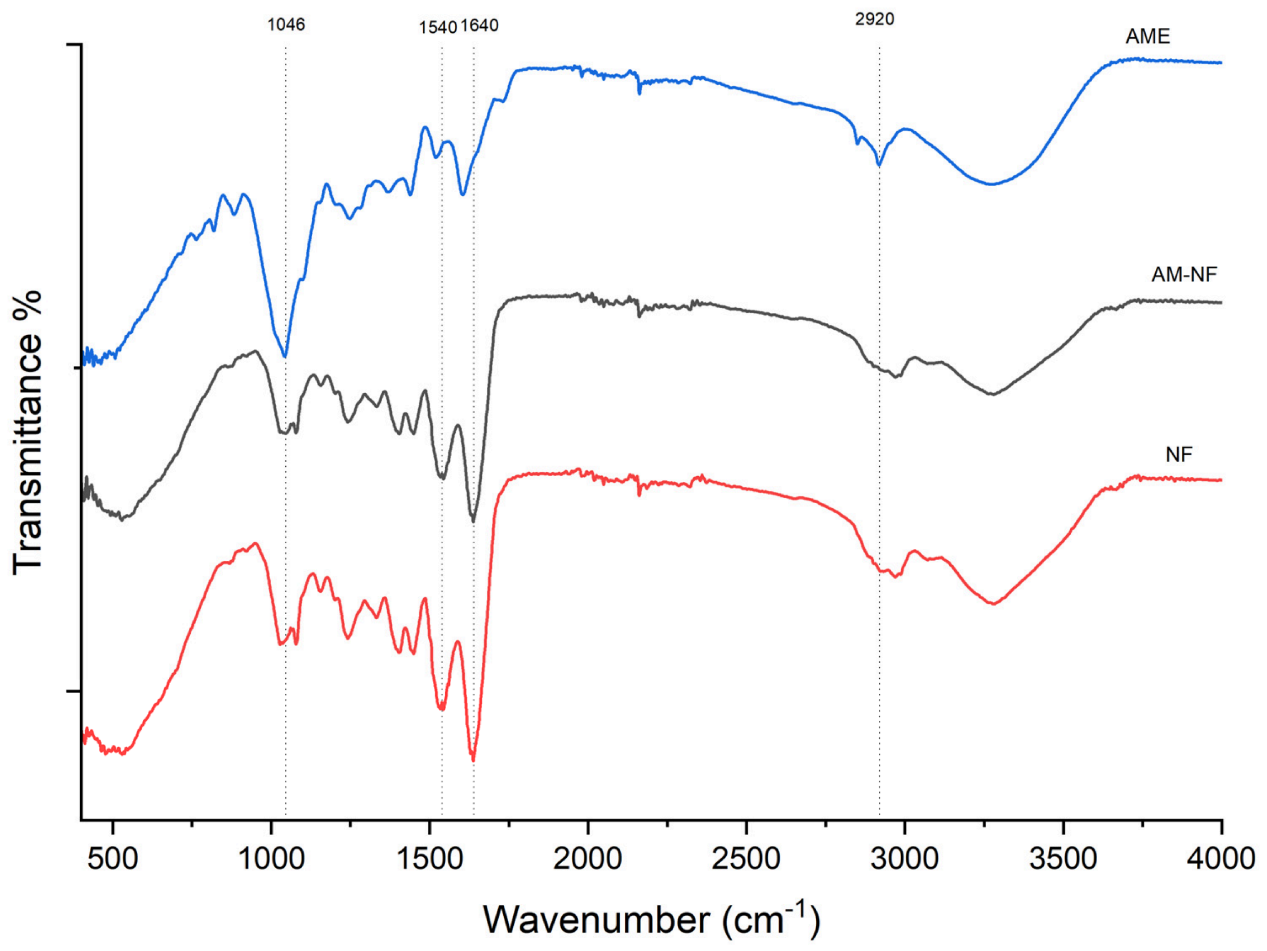
FT-IR analysis of *Aronia melanocarpa* extract, neat and extract-loaded nanofibers.

The FT-IR spectra show the functional group distributions in the AME, NF and AM-NF samples and the integration of aronia into the fiber matrix (Figure 2). A broad O–H stretching band is observed in all the samples in the 3600–3200 cm^−1^ range of the spectra. This band is associated with hydroxyl groups in the polysaccharide structure and in phenolic compounds [19]. The wider and more prominent presence of this band in the aronia sample is consistent with its high phenolic and anthocyanin contents. The bands in this region of the AME and NF spectra are widened in the AM-NF structure, which may indicate that aronia is bound to the fiber matrix and that hydrogen bonding interactions are increased. C–H stretching vibrations in the 3000–2850 cm^−1^ region are present in all samples and represent aliphatic structures. While two sharp peaks corresponding to symmetric and asymmetric C–H vibrations are observed at 2850 and 2920 cm^−1^ for AME [40], the 3000–2850 cm^−1^ range for the nanofibers appears as a broader, more diffuse region. The peak observed at approximately 1730 cm^−1^ in the AME spectrum is attributed to flavonoids and is associated with the stretching vibration of the C=O carbonyl group [19]. However, the peak observed more sharply at 1605 cm^−1^ is associated with the C=O vibrations of the glycosidic bonds and the aglycone pyranoside in aronia, which is consistent with the finding that anthocyanins contain a benzene ring [40,41]. However, sharp peaks were observed at 1640 cm^−1^ in the amide I region (C=O stretching) and at 1540 cm^−1^ in the amide II region (N–H bending) in the NF and AM-NF samples because of the protein structures of gelatin and collagen [42,43]. The 1200–1000 cm^−1^ region corresponds to the C–O–C and C–O–H stretching vibrations of polysaccharides [41]. These bands are stronger in the fiber samples. The slight change in the band profile of AM-NF compared with that of NF suggests structural modification as a result of phenolic–polymer interactions. However, the phenolic cycloether vibration is observed at 1046 cm^−1^ in AME [40]. Nevertheless, in the fingerprint region of ~1000 cm^−1^, aronia presents a more complex spectrum because of its multicomponent phenolic structure. As a general indicator, while NF presents a typical dietary fiber polysaccharide spectrum, aronia presents a phenolic and anthocyanin-rich profile. The spectrum of AM-NF reflects a combination of both structures.

### 3.4. Contact Angle

**Figure 3:**
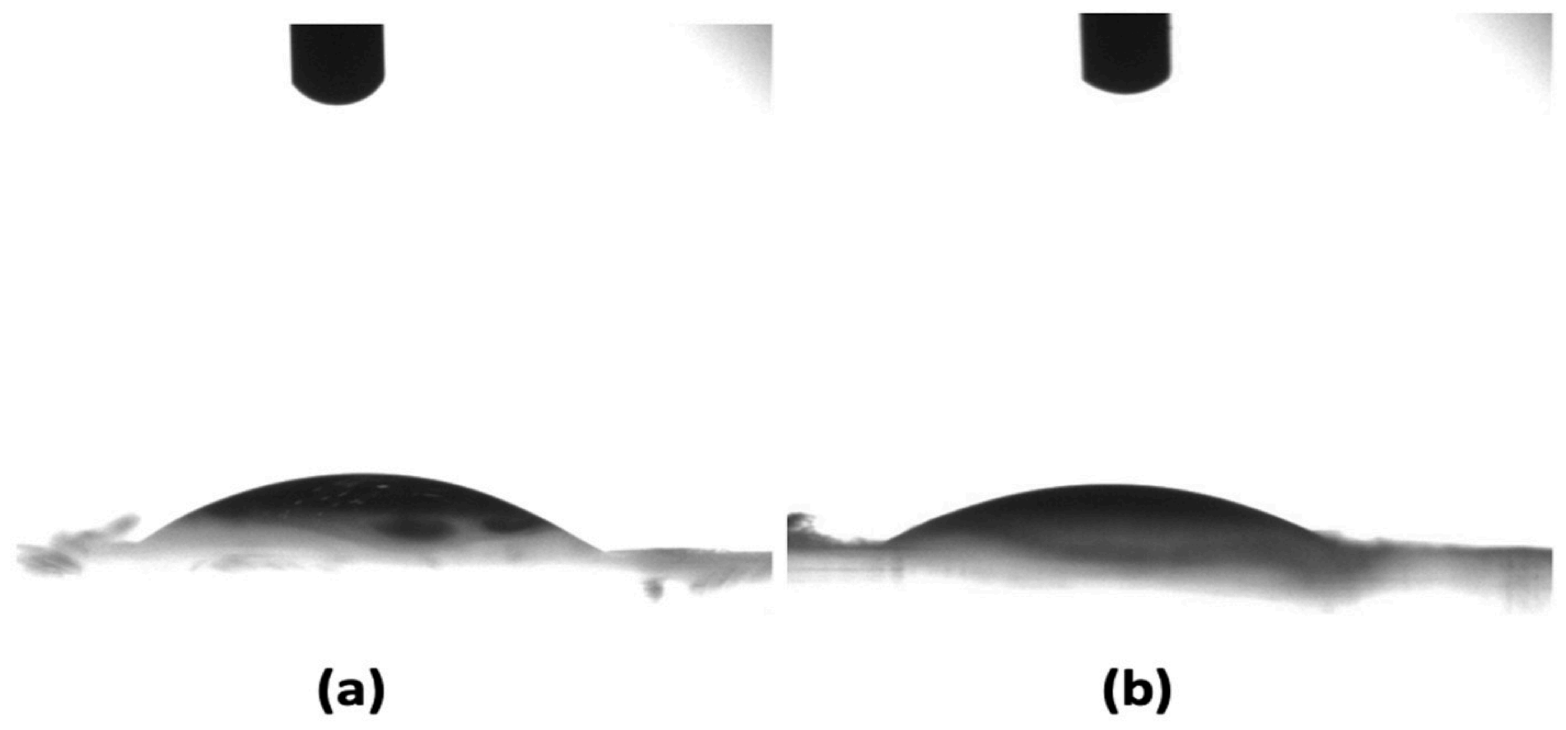
Contact angle images of (**a**) neat and (**b**) aronia-loaded nanofibers.

Water contact angle analysis was performed to observe the wettability properties of the nanofibers. The images obtained from this analysis are shown in Figure 3. The findings revealed that the contact angle of the pure NF sample was 39.71 ± 2.28°, whereas this value decreased to 28.83 ± 3.8° in the AM-NF sample (*p* < 0.05).

### 3.5. Spectrophotometric Assay

The total phenolic content and antioxidant activity of the neat and aronia-loaded nanofiber samples were determined to be 19.365 ± 2.499 mg GAE/g and 48.775 ± 2.962 mg GAE/g and 2.852 ± 0.477 mg TE/g and 7.880 ± 1.280 mg TE/g, respectively. However, the phenolic contents of the water and ethanolic extracts of aronia were 27.624 ± 5.306 and 26.046 ± 5.221 mg GAE/g, respectively, in this study, which is consistent with the literature. The total antioxidant activities of the water and ethanolic extracts were determined to be 224.292 ± 17.539 mg. TE/g and 447.696 ± 92.538 mg TE/g, respectively.

### 3.6. Analysis of the Anthocyanin Profile

Compared with blackberries, raspberries, black currants, elderberries, and blueberries, aronia has a greater anthocyanin content [44]. Despite their significant biological benefits, anthocyanins are unstable substances that are highly sensitive to temperature, light, and pH [45,46]. In this context, encapsulation is one of the methods commonly used to improve the stability of anthocyanins [44]. The anthocyanin contents of the samples are presented in Table 2. Cy3G (cyanidin-3-O-glucoside) was the most dominant fraction in both AME (12.95 mg/100 g) and AME-loaded NF (1.97 mg/100 g). Cyanidin showed the highest retention rate (17.72%) after electrospinning, followed by Cy3G (15.20%) and malvidine (12.28%). In contrast, peonidin showed the greatest reduction, decreasing approximately 45.6-fold after encapsulation, and exhibited the lowest retention rate (2.20%). No anthocyanins were detected in the empty nanofiber (NF), suggesting that encapsulation may be possible.

### 3.7. Evaluation of AME Antibacterial Activity by Growth Curve Assay

Considering the potential application of AME-loaded nanofibers as wound dressing materials, their antibacterial activity against MRSA was evaluated to investigate whether the developed formulation provides an additional biological function in addition to its antiproliferative properties. The maximal growth rates (Vmax) were extracted from the OD600–time curves by calculating the slope between consecutive time points (ΔOD600/Δt) and taking the highest value for each condition. In this way, Vmax serves as a kinetic readout of antibacterial activity, capturing how strongly the treatment perturbs exponential-phase growth. As shown in Table 3, the control culture presented a Vmax of 0.23 OD600·h^−1^. Aronia extract at 10–100 µg/mL had only minor effects on the maximal growth rate (86–101% of the control), indicating that exponential-phase kinetics were largely preserved at these concentrations. In contrast, at 500 µg/mL, the Vmax decreased to 0.12 OD600·h^−1^ (53% of the control), demonstrating a pronounced inhibition of *S. aureus* growth during the exponential phase.

**Figure 4:**
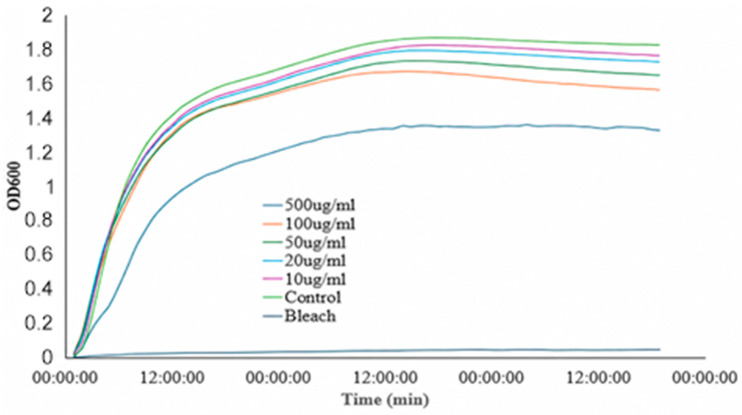
Growth curves with Vmax time markers.

Aronia extract also altered the timing of exponential growth. For the control and the 10–100 µg/mL conditions, Vmax was reached within the first 2–3 h of incubation, whereas at 500 µg/mL, the maximal slope ΔOD600/Δt occurred only after approximately 7 h (Figure 4). This delay in the onset of rapid growth suggests that high-dose aronia extract prolongs the lag phase and/or imposes an adaptation period before *S. aureus* cells can resume active division. Taken together, these kinetic parameters reveal a clear concentration-dependent pattern: low and intermediate concentrations of aronia extract (10–100 µg/mL) have minimal effects on the ΔOD600/Δt during exponential growth but modestly reduce the final OD600, which is consistent with a primarily capacity-limiting effect on the culture. In contrast, 500 µg/mL substantially decreased the Vmax, delayed the onset of exponential growth, and lowered the final OD600, indicating a strong bacteriostatic effect under these conditions. Consistently, when growth was evaluated at the final time point of the assay, cultures treated with 10–100 µg/mL aronia extract showed only modest reductions in the OD600 relative to the control (approximately 4–15%), whereas 500 µg/mL resulted in an OD600 decrease of approximately 28%. Overall, the growth curve analysis demonstrated that only the highest aronia extract concentration (500 µg/mL) had a marked inhibitory effect on *S. aureus* growth kinetics, whereas lower concentrations caused only mild restrictions in the final culture density.

### 3.8. Determination of the IC50/Proliferation Value of the Extracts from the B16F10 Cell Line

**Figure 5:**
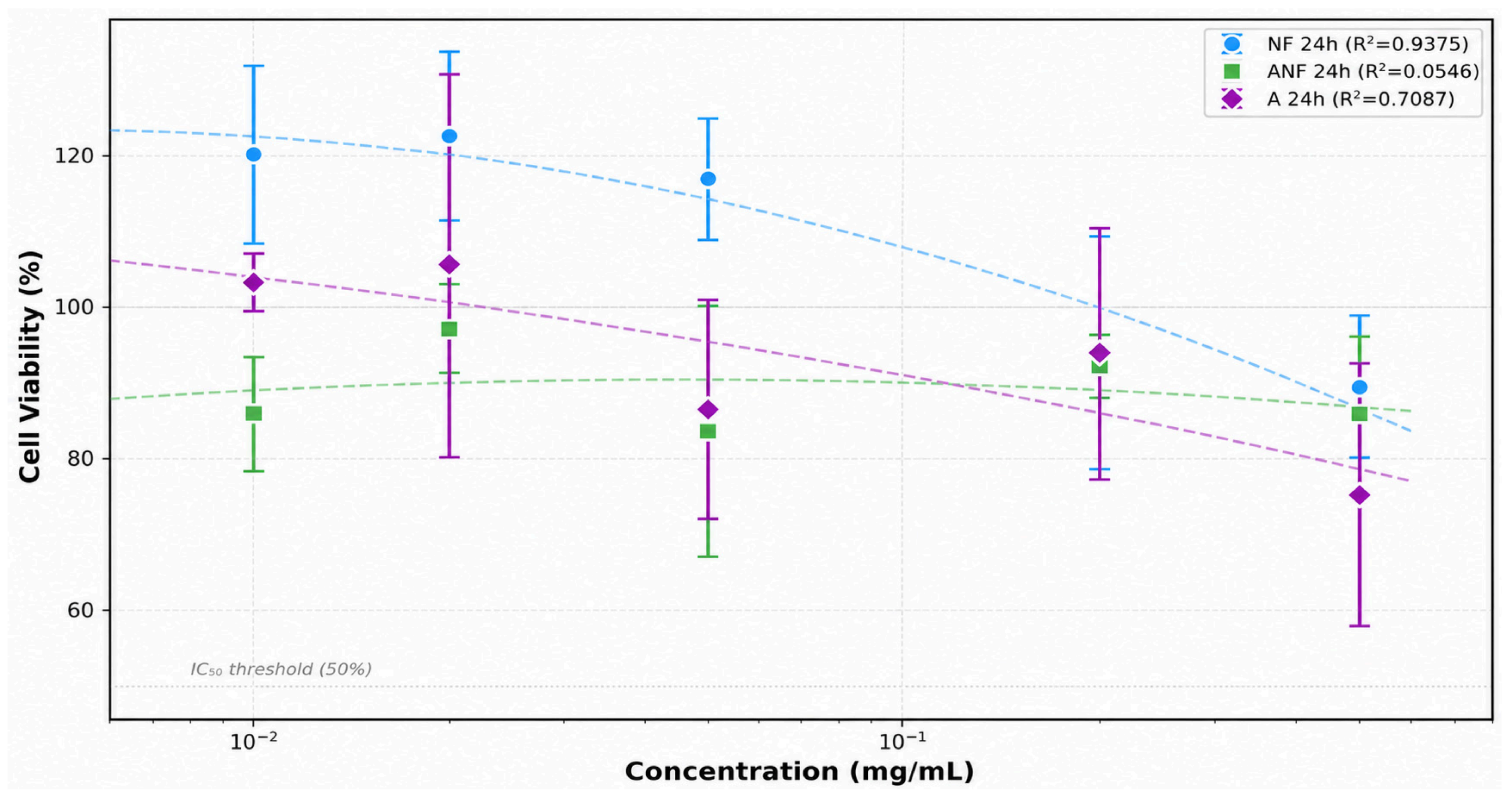
Viability of B16F10 melanoma cells following 24 h of exposure to aronia extract (A), nanofibers (NFs), or aronia-loaded nanofibers (ANFs). NF showed the clearest concentration-dependent trend in reducing cell viability within the tested concentration range, whereas ANF did not exhibit a significant dose–response relationship. The cell viability remained above the 50% threshold for all the treatments.

Figure 5 shows the results of the analyses performed to evaluate the inhibitory effects of the aronia extract, neat nanofiber, and aronia-loaded nanofiber extracts on B16F10 cells after 24 h and calculate their IC_50_ values. The aronia extract used in the experiments was prepared in DMSO at a concentration of 100 mg/mL and then diluted with DMEM at a ratio of 1:1000, adjusting the final DMSO concentration so that it did not exceed 0.1%. The estimated IC_50_ value after 24 h of treatment for the NF group was approximately 3.33 mg/mL, representing the lowest calculated IC_50_ value among the formulations tested. Group A presented an estimated IC_50_ value of approximately 8.97 mg/mL, indicating a lower apparent cytotoxic effect within the concentration range studied. In contrast, a reliable IC_50_ value could not be determined for the ANF group, as no significant concentration-dependent response was observed at 24 h. Notably, all the IC_50_ values were obtained by extrapolation beyond the experimentally tested concentration range, as the cell viability remained above 50% at all the concentrations tested. Therefore, these values should be interpreted with caution. Further studies using higher concentrations (e.g., 5–10 mg/mL) are recommended to obtain experimentally validated IC_50_ values.

### 3.9. Evaluation of Autobind Docking of Anthocyanins and the Receptor of B16F10 Melanoma Cells

It appears that the biological effects of cyanidin derivatives are not limited to general antioxidant properties but also involve specific and structure-dependent molecular interactions with target proteins. Table 4 shows that cyanidin-3-glucoside, cyanidin-3-arabinoside, cyanidin-3-galactoside, and cyanidin-3-rutinoside can occupy multiple potential binding pockets on the EGFR kinase; however, the C4 pocket stands out as a common and priority binding site for all the compounds. The fact that the C4 pocket yields the best or most consistent Vina scores despite its relatively small volume suggests that not only the pocket size but also the geometric fit of the ligand, hydrogen bonds, and polar contacts are decisive in binding. In particular, the fact that cyanidin-3-rutinoside has the strongest binding in the C4 pocket at −9.0 kcal/mol suggests that even anthocyanins with larger and more complex sugar structures can develop high affinity in a suitable microenvironment.

## 4. Discussion

Several factors, such as electrical conductivity, temperature, polymer concentration, and solvent type, affect the rheological properties of electrospinning solutions [27]. Among these parameters, viscosity is one of the most important factors determining fiber formation. Low viscosity can negatively affect fiber quality and performance by causing a decrease in polymer flow and an increase in bead formation [47,48]. The literature reports that the suitable viscosity range for electrospinning is approximately 1–200 poise (0.1–20 Pa·s), but homogeneous and bead-free nanofibers are mostly obtained in the 1–20 poise (0.1–2 Pa·s) range [49].

In this study, the addition of aronia to the feed mixture resulted in an increase in the apparent viscosity values of the mixture at the consistency index and maximum shear rate. The components contained in the aronia extract likely interact with the polymer chains, increasing the flow resistance of the solution. Indeed, previous studies have shown that increasing the aronia concentration increases the viscosity of the solution and reduces bead formation in the fibers. Similarly, in research on polyurethane nanofibers, increasing the aronia content and polymer concentration has been reported to lead to increased viscosity and the formation of larger fibers [24].

As the viscosity of the solution increases due to the addition of bioactive substances, the droplets formed during electrospinning grow larger, and their shapes transform from spherical structures to filament-like structures. This results in an increase in the diameter of the fibers obtained from electrospinning [50]. Similar trends have also been reported in nanofiber systems loaded with saffron [51], curcumin [52], bromelain [53] and probiotic bacteria [54]. In the present study, SEM images confirmed that the obtained viscosity values were sufficient for stable jet formation and homogeneous nanofiber production; furthermore, the addition of aronia supported nanofiber formation without negatively affecting the fiber morphology.

The interaction of the surface with water is increased by adding hydrophilic polar groups to the polymer components; this leads to a decrease in the contact angle and an acceleration of water absorption [55,56]. The addition of active functional groups present in aronia extract to the polymer network likely enhances the product’s interaction with water [57]. Consequently, the reduced contact angle observed in the AM-NF sample compared with that of pure NF may indicate that the addition of aronia extract effectively altered the surface properties of the material to a more hydrophilic state. The contact angle results of the study are consistent with the literature [58,59,60]. This hydrophilic property may be advantageous for applications requiring rapid swelling and efficient interaction with water-based media [55]. This hydrophilic property of AM-NF may contribute to rapid swelling and enhanced interaction with the skin. In addition, hydrophilic surfaces are desirable properties, particularly in tissue engineering, as they are crucial for wound healing applications; this property facilitates cell adhesion, migration, proliferation, and differentiation [58,59,60]. However, further biocompatibility and functional studies are needed to validate its practical applicability.

The FT-IR results demonstrated that the aronia extract was successfully integrated into the nanofiber matrix and interacted with the polymer components. The more pronounced O–H vibrations observed in the aronia extract are associated with the extract’s phenolic and anthocyanin-rich structure and are consistent with findings reported in the literature [40,41]. Some band changes and density differences observed in the nanofiber samples indicate that molecular-level interactions occur between the aronia components and the polymer chains. The literature also reports that phenolic compounds can form complex structures with polysaccharides and protein-based matrices through hydrogen bonds and other weak interactions [42]. However, the 2835–3000 cm^−1^ range becomes broader in AM-NF than in NF; this may be due to the interaction of the bonds observed at 2850 and 2920 cm^−1^ in AME with NF. In addition, the peaks observed at 1640 cm^−1^ and 1540 cm^−1^ in NF due to the protein structures of gelatin and collagen [42,43] show decreased intensity in AM-NF based on the interaction of the nanofiber with AME. The stretching vibration peak of the C-O single bond in the benzopyran structure, which appears quite distinct and sharp at ~1046 cm^−1^ in the AME spectrum [19] in AM-NF, unlike NF, appears as a shoulder in the 1025–1080 region related to the incorporation of AME. The changes in the intensity of the vibrations in the aronia-loaded nanofibers compared with those in the control group indicate that phenolic compounds are incorporated into the fiber structure and that hydrogen bond interactions are strengthened [42]. These interactions suggest that the aronia extract is homogeneously distributed in the nanofiber structure and successfully binds to the matrix. While the control nanofibers exhibited the characteristic properties of the basic polymer structure, the aronia-loaded nanofibers reflected the properties of both the polymer matrix and the aronia-derived phenolic components. This finding indicates that the aronia extract is preserved in the nanofiber structure after the electrospinning process and supports the potential of nanofibers as bioactive component carrier systems.

*Aronia melanocarpa* is considered one of the richest fruits in terms of phenolic compounds, and bioactive components such as procyanidins, anthocyanins and phenolic acids are largely responsible for the high antioxidant capacity of the fruit [46,61]. However, the phenolic profile of aronia is affected by many factors, such as genetic structure, growth conditions, maturity level, processing method and extraction conditions [62,63]. The total phenolic content of fresh aronia has been reported to vary between 690 and 2560 mg GAE/100 g FW [4]. These values can reach 298.83–364.03 mg/g under different extraction conditions [64]. Similarly, a study determined the total phenolic content in aronia samples to be 152.29 ± 2.73 mg GAE/g FW [41]. These differences reveal the effects of the plant part, solvent system and extraction parameters on the phenolic yield [24,64,65].

The distribution of phenolic compounds in different tissues of aronia is also noteworthy. A previous study reported that approximately 30% of total phenols are found in the peel, 73% of anthocyanins are concentrated in the peel, and the majority of phenolic acids (78%) are located in the fruit pulp [7]. The same study revealed that chlorogenic acids were the dominant phenolic compounds in undigested samples. Furthermore, it has been reported that not only fruit but also byproducts such as pulp and leaves contain significant amounts of phenolic compounds; the total phenolic content is reported to be 3100–6300 mg/100 g dry matter in pulp and 1946–9148 mg/100 g in leaves [61]. This situation holds significant potential for evaluating aronia byproducts as sources of functional components.

Owing to the sensitivity of phenolic compounds to oxidation, light, temperature, and storage conditions, studies on encapsulation and carrier systems for the preservation of these components have increased significantly in recent years [6]. A study achieved an encapsulation efficiency of over 88% for total phenols and anthocyanins in aronia extracts encapsulated by spray drying using maltodextrin and gum arabic, demonstrating that this approach is effective in preserving phenolic stability [4]. Similarly, studies have reported that high levels of antioxidant capacity can be preserved in different products derived from aronia [62,66].

In this study, loading aronia extract onto the nanofiber matrix resulted in an almost 2.5-fold increase in the phenolic content (approximately 2.5-fold) and antioxidant capacity (approximately 3-fold). These results demonstrate that aronia-derived phenolic compounds are largely preserved after electrospinning and successfully integrated into the nanofiber structure. Compared with those of other aronia-containing biopolymer systems, the obtained phenolic contents are quite competitive. In aronia-containing guar gum films, the total phenolic content ranges from 16.22 to 52.92 mg GAE/g film [20]. The value of 48.775 mg GAE/g obtained in this study is quite close to the upper limit of this range, indicating that the nanofiber structure can effectively carry phenolic compounds. In contrast, the total phenolic content in alginate and inulin microcapsules loaded with aronia extract was reported to be only 0.19–0.24 mg GAE/g [67]. The higher phenolic content obtained in the present study suggests that nanofibers produced by electrospinning may be an effective carrier system for the retention of bioactive compounds.

A similar trend was observed in terms of antioxidant activity. It has been reported that PVA/CS films containing aronia extract have the highest DPPH and ABTS radical scavenging capacity and that this effect is due to the presence of anthocyanins, flavonols, and proanthocyanidins [17]. Further studies reported that the films showed approximately 80% radical scavenging activity in the DPPH test and close to 99% radical scavenging activity in the ABTS test [18]. The increase in antioxidant activity observed in the present study similarly demonstrated that aronia-derived phenolic compounds retain their functional properties. The preservation of the characteristic functional groups of phenolic structures via FT-IR analyses also supports this result. Similar results were observed in terms of antioxidant activity. It has been reported that PVA/CS films containing aronia extract have the highest DPPH and ABTS radical scavenging capacity and that this effect is due to the presence of anthocyanins, flavonols, and proanthocyanidins [17]. Further studies reported that the films showed approximately 80% radical scavenging activity in the DPPH test and close to 99% radical scavenging activity in the ABTS test [18]. The increase in antioxidant activity observed in the present study similarly demonstrated that aronia-derived phenolic compounds retain their functional properties. The preservation of the characteristic functional groups of phenolic structures via FT-IR analyses also supports this result.

In this study, the loading of aronia extract onto the nanofiber matrix not only ensured the preservation of phenolic compounds but also contributed to a significant increase in antioxidant capacity. The porous structure with a high surface area obtained by electrospinning supported the homogeneous distribution of phenolic compounds in the matrix and helped preserve functional properties. Therefore, aronia-loaded nanofibers can be considered promising natural antioxidant carriers for active food packaging, functional foods, and nutraceutical applications.

The predominant anthocyanin compounds in the samples were identified as cyanidin-3-O-glucoside and pelargonidin-3-O-glucoside (Pg3G), cyanin, and malvidin; however, in the literature, the predominant compounds in aronia are listed as cyanidin-3-O-galactoside, cyanidin-3-O-glucoside, cyanidin-3-O-arabinoside, and cyanidin-3-O-xyloside [46,68,69,70,71]. However, the levels of anthocyanins can vary depending on numerous parameters, such as the type of anthocyanin, planting location, climate, degree of fruit ripeness, genetic factors, processing, storage conditions and even extraction procedure, and the analytical method used to determine the anthocyanin profile [69,72,73]. The extraction conditions of the AME used in this study, the analytical method, and the existence and type of standard selected for the anthocyanin profile all influenced the anthocyanin compounds of the samples. HPLC analysis showed that Cy3G (cyanidin-3-O-glucoside) remained the dominant anthocyanin after electrospinning, indicating that the main bioactive component of AME is effectively protected within the nanofiber matrix. Although all anthocyanins were reduced after encapsulation, cyanidin and Cy3G showed the highest retention rate. This suggests that the compounds have greater stability than other anthocyanins during electrospinning and subsequent extraction. In contrast, the observed significant reduction for peonidin may reflect higher degradation susceptibility or lower encapsulation efficiency under production conditions. The absence of detectable anthocyanins in the blank nanofibers further confirms that the integrated AME is the sole source of these bioactive compounds. Importantly, the predominance and protection of cyanidin-derived anthocyanins may at least partially explain the enhanced biological performance of AME-loaded nanofibers. This interpretation is supported by molecular docking results revealing a strong binding affinity (−9.0 kcal/mol) of cyanidin-3-O-rutinoside to the EGFR kinase domain, suggesting that cyanidin-based anthocyanins may contribute to concentration-dependent antiproliferative activity through modulation of EGFR-related signaling pathways.

This study demonstrated that extracts can inhibit the growth of *Escherichia coli* (*E. coli*) and *Staphylococcus aureus* (*S. aureus*). Researchers have used molecular docking to investigate potential mechanisms associated with antibacterial activity, focusing on essential proteins for survival and colonization in these bacteria [74]. These results are consistent with those of previous studies demonstrating the strong antimicrobial potential of aronia-derived phenolic compounds. The ethanol extract from *Aronia melanocarpa* pulp has been reported to exhibit over 99% antibacterial activity, particularly against *Escherichia coli* and *Staphylococcus aureus*. More importantly, this effect is maintained not only in free extracts but also when they are integrated into different biomaterial systems. For example, textile materials functionalized with aronia pulp extract exhibited over 75% and 80% antibacterial activity against *E. coli* and *S. aureus*, respectively, whereas starch/gelatin-based hydrogel systems containing aronia extract achieved over 70% inhibition of *E. coli* and over 97% inhibition of *S. aureus* [8]. These findings demonstrate that aronia-derived polyphenols can maintain their biological activity even when loaded onto carrier systems. These antibacterial findings further support the potential biomedical application of AME-loaded nanofibers. Since wound dressings are expected not only to provide structural support but also to reduce bacterial contamination, the observed inhibition of MRSA growth represents an additional functional advantage of the developed material.

The antimicrobial efficacy of aronia extracts also depends on the extraction method and solvent type used. In the literature, methanolic extracts have been reported to have greater antimicrobial activity than ethanol- or acetone-based extracts do [3,75]. This effect is largely attributed to bioactive compounds such as phenolic acids, flavonoids, and anthocyanins present in the extract, and these compounds alter cell membrane permeability by creating oxidative stress in microorganisms [75,76]. Similar antimicrobial effects have been observed in food systems containing aronia. For example, it has been reported that honey samples enriched with aronia show increased antibacterial activity against both Gram-positive and Gram-negative bacteria, and particularly with the addition of 4% aronia, significant bacterial growth inhibition is achieved [77]. This effect is thought to be due to the synergistic interaction of aronia polyphenols with the natural antimicrobial properties of honey. Overall, the growth curve data obtained in this study are consistent with the bacteriostatic effects of aronia extracts reported in the literature. In particular, the decrease in Vmax and the prolongation of the lag phase observed at high concentrations indicate that aronia polyphenols can significantly alter bacterial growth kinetics.

In the present study, aronia-loaded nanofibers affected microbial growth kinetics, particularly by reducing the maximum growth rate (Vmax) and prolonging the lag phase at high concentrations. These findings suggest that phenolic compounds remain biologically active even after electrospinning and that the nanofiber matrix can effectively transport these compounds. Indeed, the high total phenolic content and antioxidant activity values determined in our study support the preservation of phenolic compounds, which are fundamental to antimicrobial activity, within the nanofiber structure. The effects of aronia-derived phenolics are not limited solely to the suppression of pathogenic bacteria. Recent studies have shown that polyphenol-rich extracts obtained from fruit pulps can have selective effects on the microbiota. It has been reported that extracts obtained from aronia, blackberry, and raspberry pulp suppress *S. aureus* growth and support microbial balance when used in combination with *Staphylococcus epidermidis*, a beneficial skin bacterium [78]. These findings suggest that aronia phenolics may possess not only antimicrobial but also microbiota-regulating properties.

The antimicrobial effects of phenolic compounds are explained by more complex mechanisms beyond direct growth inhibition. Various plant phenolics have been shown to suppress bacterial biofilm formation and, in some cases, inhibit efflux pump systems associated with antibiotic resistance [79]. In particular, phenolic compounds such as apigenin-7-O-glucoside and arbutin have been reported to significantly reduce biofilm formation in *E. coli* and methicillin-resistant *S. aureus* (MRSA) strains. These results demonstrate that phenolic compounds have multifaceted mechanisms of action on bacteria and can affect not only cell proliferation but also the environmental adaptation processes of bacteria.

Aronia compounds can exhibit anti-inflammatory and antitumor effects due to their strong antioxidant properties. Chokeberry polyphenol extract has been reported to inhibit the proliferation of human liver cancer cells and to have a protective effect on pancreatic β-cells [5]. Aronia leaf extract significantly reduces the migration and invasion capabilities of colorectal SW-480 cells [80].The methanolic extract of *Aronia melanocarpa* has been reported to have antitumor activity in a 4T1 mouse model of breast cancer and in MCF-7 and MDA-MB-231 human breast cancer cells. Aronia extracts have been reported to reduce tumor volume, increase apoptosis, and suppress the expression of tumor stem cell markers, suggesting that aronia-derived bioactive compounds may be potential therapeutic agents for cancer treatment [81,82].

Aronia polyphenols have been reported to suppress proliferation in HeLa, colorectal, and liver cancer cells and to exhibit chemopreventive effects in mouse models exposed to cigarette smoke [83]. In general, *A. melanocarpa* extracts have been shown to inhibit cancer cell growth and exhibit antitumor activity by modulating mechanisms associated with oxidative stress [82]. Furthermore, the effects of some flavonoid compounds on melanogenesis have also been investigated. For example, fraxinol has been shown to increase melanin content and tyrosinase activity in B16F10 melanoma cells, increase the mRNA expression of melanogenic enzymes, and increase CREB phosphorylation [83]. Similarly, the flavonoid morin has been shown to increase melanin biosynthesis in B16F10 mouse melanoma cells without causing cytotoxicity, and this effect is achieved through the activation of MAPK signaling pathways (ERK and p38) [84].

Recent advancements in sustainable biomaterials are increasingly focusing on the design and integration of bio-based platforms that combine renewable resources with functional bioactive components to improve biomedical performance. One study demonstrated that biomimetic holocellulose materials inspired by spider silk can simultaneously enhance mechanical strength and toughness through nature-inspired hierarchical architectures for sustainable material design [85]. Similarly, bio-based polyurethane foams derived from rapeseed oil loaded with silver nanoparticles have been developed as wound dressings that improve water absorption, antibacterial activity, cytocompatibility, and wound healing [86]. Building on this concept, the present study utilizes electrospinning-produced gelatin–collagen nanofibers as a scaffold mimicking a naturally occurring extracellular matrix, while incorporating AME a polyphenol-rich sustainable agricultural-industrial byproduct, to provide antioxidant, antimicrobial, and antiproliferative activities. All of these studies demonstrate the ongoing shift from petroleum-derived or fully synthetic materials to multifunctional, bio-based, and environmentally sustainable wound healing platforms. Although different biomaterial platforms were used, the common goal of all three studies is to develop sustainable, bio-based multifunctional materials inspired by natural systems to enhance biomedical performance through improved structural and biological functionality.

Cyanidin derivatives, which are among the main anthocyanins of aronia, may exert anticancer effects [87] particularly through EGFR-mediated signaling pathways [66]. Cyanidin-3-O-glucoside (C3G) inhibits the EGFR/AKT signaling pathway by binding to ERα36 in EGFR-positive triple-negative breast cancer (TNBC) cells, increasing EGFR degradation, and stimulating apoptosis, thereby inhibiting tumor growth [88]. Similarly, in UVB-damaged human keratinocytes, C3G has been shown to reduce EGFR phosphorylation, suppress the p38, ERK, and JNK pathways, and protect cells against UV-induced damage by reducing ROS and COX-2 formation [89]. However, in melanoma models, the effect of C3G is mediated mainly by ERβ, independent of EGFR; it has been reported to arrest the cell cycle in the G2/M phase, increase apoptosis, and suppress tumor growth [90]. It has also been shown to promote differentiation and reduce proliferation in human melanoma cells [91].

Molecular docking studies on the interaction of aronia anthocyanins with EGFR have also yielded noteworthy results. Cyanidin aglycone strongly inhibits EGFR tyrosine kinase activity in A431 carcinoma cells overexpressing EGFR, whereas cyanidin-3-galactoside does not significantly inhibit EGFR activity at concentrations up to 100 µM [92]. These findings suggest that the sugar group in anthocyanins may be a determining factor in their biological activity. On the other hand, network pharmacology and molecular docking analyses have predicted that cyanidin-3-glucoside, cyanidin-3-galactoside, and cyanidin-3-rutinoside may interact with EGFR; in particular, cyanidin-3-rutinoside has been reported to have one of the highest binding affinities [68,69]. Similarly, docking analyses performed in this study revealed that cyanidin-3-rutinoside exhibited stronger binding than cyanidin-3-arabinoside, cyanidin-3-galactoside and cyanidin-3-glucoside did.

Taken together, these findings suggest that the aronia and cyanidin derivatives found in aronia pulp may target different molecular mechanisms that can suppress the proliferation of cancer cells. Although the current evidence for cyanidin-3-rutinoside is largely limited to in silico studies, both the high EGFR binding affinity reported in the literature and the docking results obtained in this study suggest that this compound may play an important role in aronia-derived anticancer activity. In particular, considering the cyanidin derivative-rich structure of aronia pulp, the observed antiproliferative effects of AME may be related to the modulation of the EGFR/AKT and ERβ-related signaling pathways.

Aronia pulp extract has been converted into suitable antioxidant and antibacterial hydrogels for wound care, and several similar plant extracts have been successfully electrospun into nanofiber dressings with good mechanical properties and enhanced healing. These findings suggest that aronia pulp is a strong candidate for future nanofiber-based wound or transdermal dressings, although such systems are not yet reported here. In the literature, nanofiber systems enriched with various plant extracts have been shown to promote wound healing. Nanofibers containing pomegranate peel and pomegranate flower extract exhibited strong antioxidant and antibacterial properties while accelerating wound closure, whereas nanofibers loaded with *Melia dubia* leaf extract promoted tissue regeneration by increasing fibroblast proliferation. Similarly, nanofibers containing *Momordica charantia* and *Acacia* extracts have been reported to contribute positively to the wound healing process because of their biocompatible structures, controlled release properties, and broad-spectrum antimicrobial activities. These findings demonstrate that plant extract-loaded nanofibers are promising biomaterials for wound dressing applications. These findings demonstrate that phenolic compounds obtained from aronia pulp not only exhibit strong antioxidant properties but also remain stable within biomaterial systems. Therefore, aronia pulp can be considered a sustainable and high value-added bioactive source for advanced carrier systems such as nanofibers for the preservation and controlled transport of phenolic compounds.

### Study Limitations

The present study evaluated B16F10 cell viability within a limited concentration range and during a single exposure period. Because cell viability remained above 50% at all tested concentrations, definitive IC_50_ values could not be experimentally established. Therefore, the current findings should be regarded as preliminary evidence of concentration-dependent effects rather than quantitative measures of cytotoxic potency. Another limitation of the present study is that sample-specific blank wells containing aronia extract without cells were not included during the MTT assay. Consequently, the potential contribution of the intrinsic color of the phenolic-rich extract to the absorbance measurements cannot be entirely ruled out. Future studies will incorporate sample-specific blank correction and complementary cell viability assays to minimize potential color interference and further validate the cytotoxicity findings. In addition, broader dose ranges, multiple exposure times, apoptosis assays, and release kinetics studies will provide a more comprehensive evaluation of the anticancer potential of AME-loaded nanofibers. One of the other limitations, nevertheless, is that the inclusion of a normal skin cell line would provide valuable information regarding the selectivity and cytocompatibility of the developed nanofibers. This limitation has now been acknowledged in the revised manuscript. Another limitation of the present study is that the cytotoxicity assessment was performed only in the B16F10 melanoma cell line, which is a well-established model for the preliminary evaluation of antiproliferative activity. Therefore, the present findings do not allow conclusions regarding selective anticancer activity or cytocompatibility toward normal skin cells. Future studies should include normal skin cell models, such as L929 fibroblasts or HaCaT keratinocytes, to evaluate the selectivity and safety of the developed nanofibers. In addition, molecular docking analysis provides only preliminary computational evidence of a potential interaction between anthocyanin derivatives and the EGFR kinase domain. These findings should not be interpreted as confirmation of an EGFR-mediated antiproliferative mechanism. Future studies involving EGFR expression and phosphorylation analyses, apoptosis assays, ROS measurements, and cell cycle analysis are needed to experimentally validate the proposed mechanism. Furthermore, the antibacterial activity was evaluated only against the methicillin-resistant *Staphylococcus aureus* (MRSA) substrain COL. Although this pathogen represents one of the most clinically relevant bacteria associated with wound infections, future studies should investigate the antimicrobial efficacy of the developed nanofibers against a broader panel of Gram-positive and Gram-negative bacterial strains to comprehensively assess their antimicrobial potential.

## 5. Conclusions

This study demonstrated that aronia extract can be successfully integrated into functional nanofiber systems as a sustainable and high-value bioactive source. These findings show that phenolic compounds from aronia can be preserved within the nanofiber structure and maintain their biological activity. Significant increases in antioxidant capacity were observed with the addition of aronia, and it was determined that cancer cell proliferation was suppressed and that microbial growth was limited. These results demonstrate that aronia is not only a food processing byproduct but also a valuable source of bioactive components. Although numerous studies exist in the literature on aronia and the phenolic composition and biological activities of aronia, studies on the behavior of these compounds in nanofiber-based systems and the preservation of their biological activity are still limited. The molecular mechanisms underlying the antiproliferative effects of phenolic compounds, particularly those derived from aronia pods, and how this activity is modulated by carrier systems have not been fully elucidated. Furthermore, while the vast majority of current studies focus on antioxidant properties, multifunctional systems that evaluate antioxidant, antimicrobial, and antiproliferative effects simultaneously are quite limited. These findings provide preliminary evidence that AME-loaded nanofibers can influence melanoma cell viability while preserving the physicochemical advantages of the electrospun scaffold. However, further studies using expanded dose–response designs are needed to establish their antiproliferative potency and determine reliable IC_50_ values.

The results obtained in this study demonstrate that aronia pod-loaded nanofibers have significant potential for the development of naturally derived and multifunctional biomaterials. The combination of high antioxidant activity, bacteriostatic effects, and hydrophilic surface properties suggests that these systems could be used in biomedical applications, particularly in wound dressings, skin regeneration, and transdermal bioactive compound delivery, with further studies. Furthermore, the antiproliferative effect observed in melanoma cells and the high affinity of cyanidin glycosides, the main components of aronia, with the EGFR receptor in molecular docking analyses suggest that these compounds may have the potential to modulate cancer-related signaling pathways. However, further research in different cancer models, animal studies, and advanced biological systems is needed to confirm these findings. In future studies, it will be important to examine the release kinetics of the active compounds in detail, determine their gastrointestinal and transdermal bioavailability profiles, elucidate their cellular signaling pathways, and evaluate their in vivo biosafety and therapeutic efficacy. In addition, the evaluation of aronia pulp as an agro-industrial byproduct offers significant opportunities in terms of a circular bioeconomy and sustainable biomaterial production. In conclusion, aronia pulp-based electrospun nanofiber systems are promising platforms for the conservation, controlled release, and enhancement of the biological activity of natural phenolic compounds, providing a strong foundation for future translational research.

## Figures and Tables

**Table 1:** Feed solution composition and properties with respect to polymers.

Sample	PC	Solution	AME *	C (mS/cm)	Κ (Pa·sn)	*n* (−)	MSR	η (Pa·s) at MSR
NF	Gel + Col (20% + 20%, *w*/*v*)	AA (50%, *v*/*v*)	—	2.87 ± 0.15	0.66 ± 0.066 ^a^	0.97 ± 0.02	1.41 ± 0.01	0.66 ± 0.06 ^a^
AM-NF			10%	2.69 ± 0.07	1.64 ± 0.34 ^b^	0.84 ± 0.03	1.46 ± 0.02	1.54 ± 0.29 ^b^

* with respect to polymer. Different letters indicate significant difference between samples at *p* ≤ 0.05. AA: acetic acid; PC: polymer concentration; Gel: gelatin; Col: collagen; C: Conductivity. Different letters (a, b) indicate significant differences (*p* < 0.05).

**Table 2:** Anthocyanin profile of samples.

Anthocyanin Profile	AME	AME-Loaded NF	NF	F Value	*p* Value
Cyanidin	2.65984 ± 0.00111	0.47145 ± 0.00048	Nd	9,838,299	6.20 × 10^−14^
Delphinidin	1.03855 ± 0.00107	0.04341 ± 0.00002	Nd	2,579,719	9.02 × 10^−13^
Cy3G	12.95497 ± 0.01548	1.96924 ± 0.00133	Nd	1,499,658	2.67 × 10^−12^
Cy-3-rut	1.28074 ± 0.00197	0.04513 ± 0.00003	Nd	1,180,139	4.31 × 10^−12^
Pg3G	3.67037 ± 0.00232	0.32330 ± 0.00016	Nd	6,212,176	1.55 × 10^−13^
Peonidin	0.72890 ± 0.00093	0.01600 ± 0.00002	Nd	1,745,683	1.97 × 10^−12^
Malvidin	2.00539 ± 0.00224	0.24636 ± 0.00008	Nd	1,850,664	1.75 × 10^−12^

One-way ANOVA was applied between the AME and AME-loaded NF groups for each anthocyanin (*n* = 3/group). Triplicate values were simulated based on the instrument RSD (≈0.1%). AME: *Aronia melanocarpa* extract, AME-loaded NF: *Aronia melanocarpa* extract Nano Fiber, Cy3G: Cyanidin-3-O-glucoside, Cy-3-rut: Cyanidin-3-O-rutinoside, Nd: Not detected, NF: Nano Fiber, Pg3G: Pelargonidin-3-O-glucoside.

**Table 3:** Effect of different doses on bacterial growth rate (Vmax, OD600/h).

Dosage	Vmax (OD600/h)	Fraction to Control
500 µg/mL	0.121	%53 (0.53×)
100 µg/mL	0.196	%86 (0.86×)
50 µg/mL	0.208	%92 (0.92×)
20 µg/mL	0.229	%101 (1.01×)
10 µg/mL	0.226	%99 (0.99×)
Control	0.227	%100

**Table 4:** Structure-based blind docking.

CurPocket ID	Vina Score	Cavity Volume (Å3)	Center (x, y, z)	Docking Size (x, y, z)	Auto Blind Docking
EGFR-C3G	−7.7	277	53, −1, −23	22, 22, 22	** 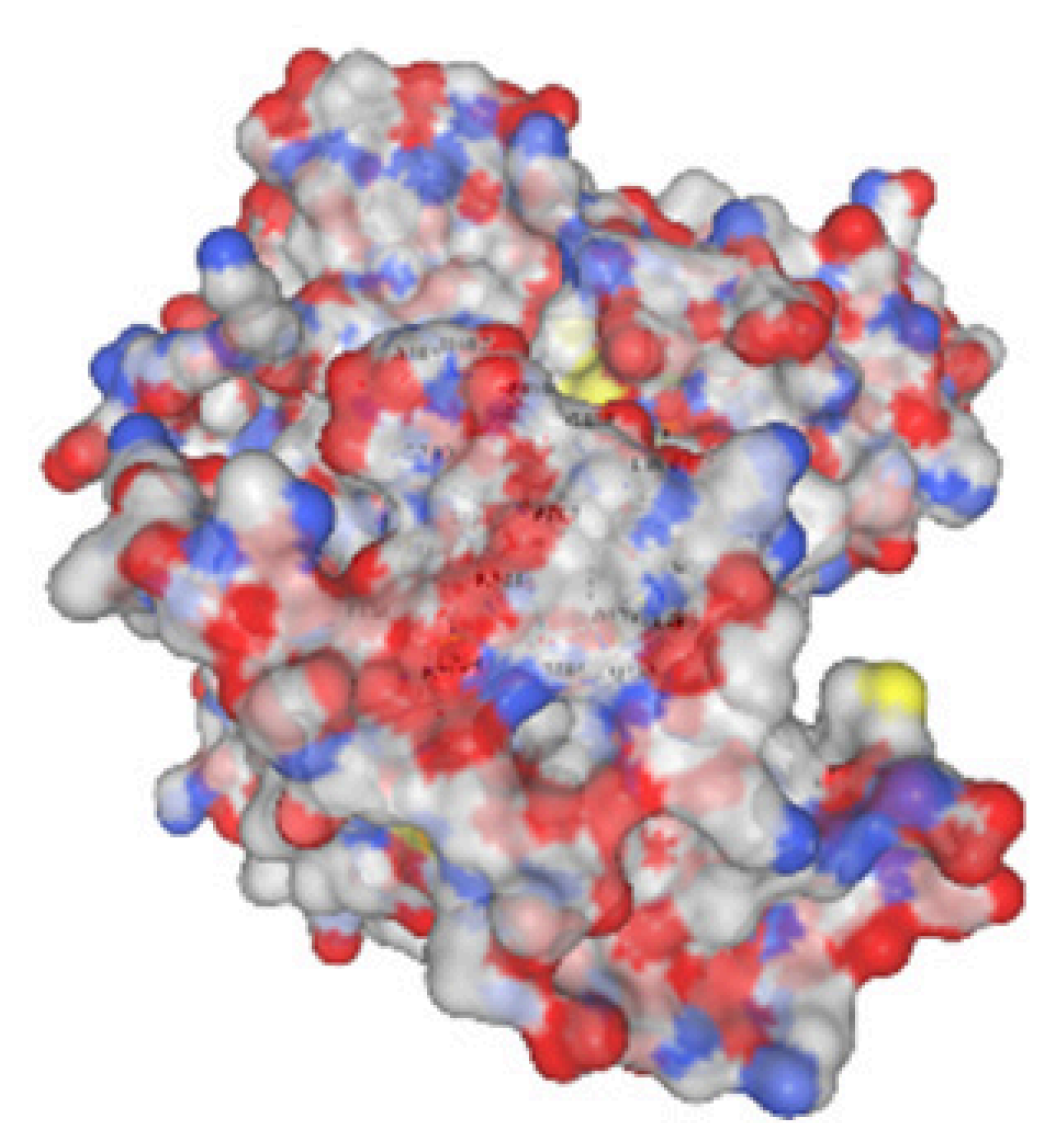 **
EGFR-C3A	−8.0	277	53, −1, −23	22, 22, 22	** 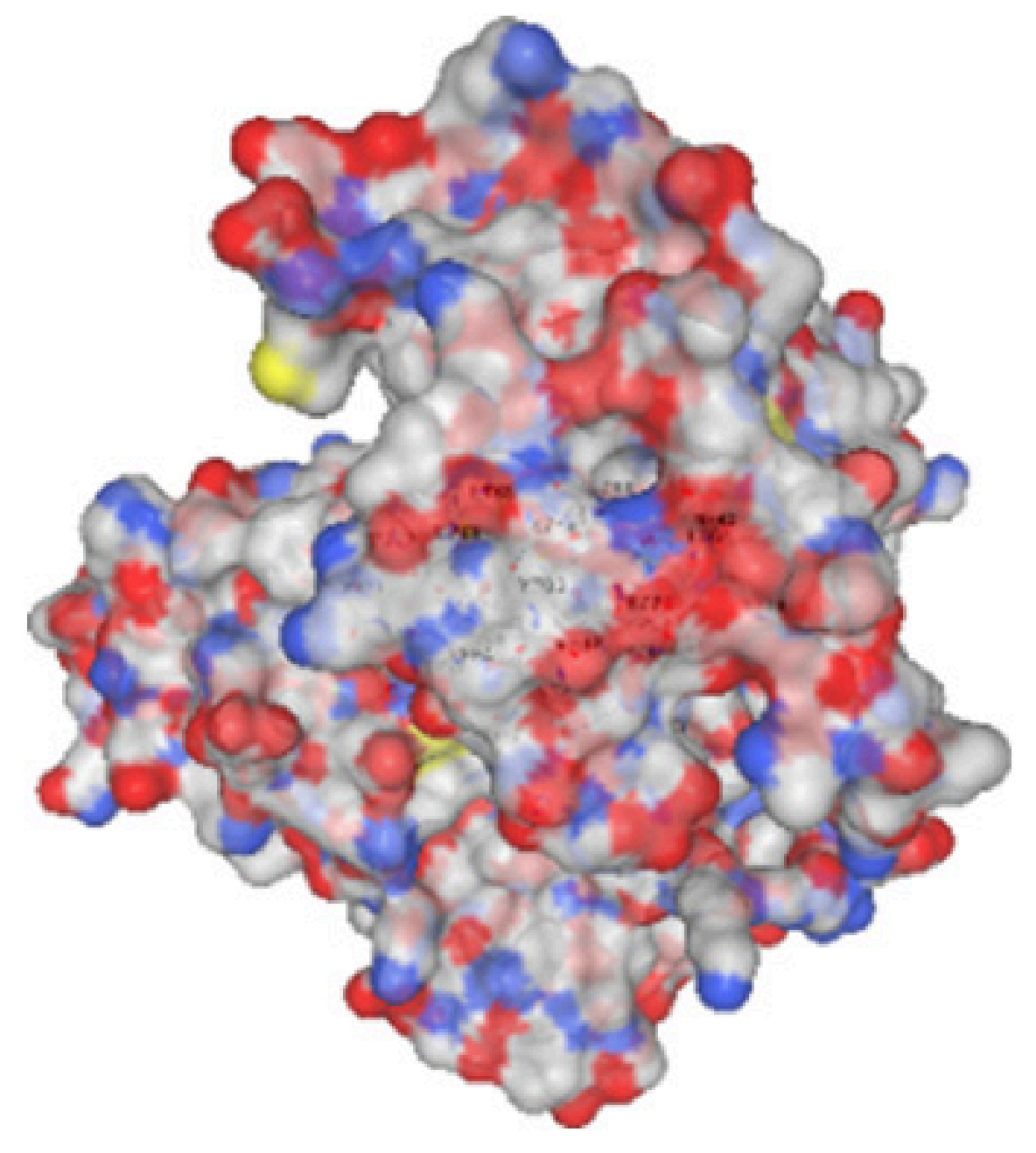 **
EGFR-Cy3Gal	−7.9	277	53, −1, −23	22, 22, 22	** 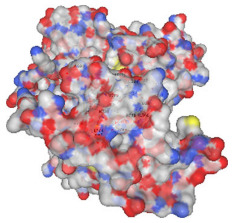 **
EGFR-C3R	−9.0	277	53, −1, −23	24, 24, 24	** 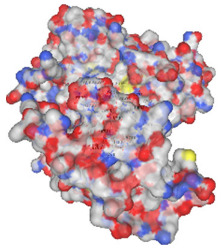 **

C3G: Cyanidin-3-O-glucoside, C3A: Cyanidin-3-O-arabinoside, Cy3G: Cyanidin-3-O-galactoside, C3R: Cyanidin-3-O-rutinoside, EGFR: epidermal growth factor receptor.

## Data Availability

The data presented in this study are available upon request from the corresponding authors.

## Abbreviations

The following abbreviations are used in this manuscript:

ABTS	2,2′-Azino-bis(3-ethylbenzothiazoline-6-sulfonic acid) diammonium salt
AM-NF	Aronia-loaded nanofiber
AME	*Aronia melanocarpa* extract
B16F10	Cutaneous melanoma cell
C3G	Cyanidin-3-O-glucoside
CFU	Colony Forming Unit
CREB	Cyclic AMP-Response Element Binding Protein
CUPRAC	Copper (II)-reducing antioxidant capacity
DMEM	Dulbecco’s Modified Eagle’s Medium
DMSO	Dimethyl sulfoxide
DPPH	2,2-Diphenyl-1-picrylhydrazyl
EGFR	Epidermal growth factor receptor
FTIR	Fourier Transform Infrared Spectroscopy
GAE	Gallic acid equivalent
IC_50_	Half maximal inhibitory concentration
MAPK	Mitogen-activated protein kinase
MSR	Maximum shear rate
MTT	3-(4,5-Dimethylthiazol-2-yl)-2,5-Diphenyltetrazolium Bromide
NF	Nanofiber
OD600/h	Optical density at 600 nm
PDB	Protein Data Bank
RNS	Reactive nitrogen species
ROS	Reactive oxygen species
SEM	Scanning electron microscopy
TAC	Total anthocyanin content
TE	Trolox equivalent
TNBC	Triple-negative breast cancer
TPC	Total phenolic content
